# A Non-Contact Method for Detecting and Evaluating the Non-Motor Use of Sidewalks Based on Three-Dimensional Pavement Morphology Analysis

**DOI:** 10.3390/s25061721

**Published:** 2025-03-10

**Authors:** Shengchuan Jiang, Hui Wang, Wenruo Fan, Min Chi, Xun Zhang, Jinlong Ma

**Affiliations:** 1 Department of Traffic Engineering, Business School, University of Shanghai for Science and Technology, Shanghai 200090, China; jsc@usst.edu.cn (S.J.); 2135070920@st.usst.edu.cn (J.M.); 2 School of Civil Engineering, Chongqing University, Chongqing 400045, China; 15080868861@163.com (W.F.); 202216131139t@stu.cqu.edu.cn (M.C.); jackyzhang@cqu.edu.cn (X.Z.)

**Keywords:** sidewalk safety, skid resistance, 3D morphometry, non-contact detection, micro-texture, pavement drainage

## Abstract

This study proposes a non-contact framework for evaluating the skid resistance of shared roadside pavements to improve cyclist and pedestrian safety. By integrating a friction tester and a laser scanner, we synchronize high-resolution three-dimensional (3D) surface texture characterization with friction coefficient measurements under dry and wet conditions. Key metrics—including fractal dimension (*FD*), macro/micro-texture depth density (*HL_TX_* and *WL_TX_*), mean texture depth (*MTD*), and joint dimensions—were derived from 3D laser scans. A hierarchical regression analysis was employed to prioritize the influence of texture and joint parameters on skid resistance across environmental conditions. Combined with material types (brick, tile, and stone) and drainage performance, these metrics are systematically analyzed to quantify their correlations with skid resistance. Results indicate that raised macro-textures and high *FD* (>2.5) significantly enhance dry-condition skid resistance, whereas recessed textures degrade performance. The hierarchical model further reveals that *FD* and *MTD* dominate dry friction (*β* = 0.61 and −0.53, respectively), while micro-texture density (*WL_TX_*) and seam depth are critical predictors of wet skid resistance (*β* = −0.76 and 0.31). In wet environments, skid resistance is dominated by micro-texture density (*WL_TX_* < 3500) and macro-texture-driven water displacement, with higher *WL_TX_* values indicating denser micro-textures that impede drainage. The study validates that non-contact laser scanning enables efficient mapping of critical texture data (e.g., pore connectivity, joint depth ≥0.25 mm) and friction properties, supporting rapid large-scale pavement assessments. These findings establish a data-driven linkage between measurable surface indicators (texture, morphometry, drainage) and skid resistance, offering a practical foundation for proactive sidewalk safety management, especially in high-risk areas. Future work should focus on refining predictive models through multi-sensor fusion and standardized design guidelines.

## 1. Introduction

### 1.1. Background

Sidewalks are one of the most influential infrastructures for human beings and constitute an indispensable part of community life [1,2,3]. However, injuries occurring on sidewalks are highly common, and the expanding elderly population is contributing to this issue [4,5]. In urban areas, in some places where there is not enough space, it is common for non-motorized paths and footpaths to be shared, which makes accidents even more likely to happen. The increasing number of e-bicycles makes this situation even worse, which should warrant attention [6]. Nevertheless, sidewalk accidents are frequently not reported to the police; thus, such accidents are difficult to find in traffic accident statistics [7,8,9]. Sidewalk safety issues typically arise from factors such as shared space [10,11], slippery road surfaces [12,13,14,15,16,17], pollutants [18], obstacles [19], and low temperatures (ice and snow) [20]. A five-year data collection showed that the percentage of bicycle accidents was caused by falls (50%), road defects (36%), and accidents caused by collisions with motor vehicles (29%) [21]. According to the literature review, the proportion of injured cyclists involved in bicycle accidents (17–85%) varies considerably between studies and data sources, with the most (>50%) typical causes of bicycle accidents being skidding or loss of control [22].

Some of the causes of these injuries are due to poor management by authorities, while others are due to unavoidable natural conditions and physical defects in the roads themselves. Managers can take the necessary steps to reduce the occurrence of cause-specific safety incidents [10]. On the other hand, those accidents that are predominantly slips, falls, and loss of control of a single bicycle require comprehensive analysis at the management level of the pavement and walking/riding path system [12,13,14,15]. The diverse composition of pavement materials and pavement geometries in existing urban road construction, and the need for ’attention’ due to changes in pavement materials and patterns that may not be detected in time by pedestrians or cyclists, pose a challenge to the continuity of the traveled pavement, as well as a potential risk to pedestrian and cyclist safety, and this risk is exacerbated by the emergence of e-pedestrianism and e-bicycling, but there is a lack of systematic research and regulatory standards.

### 1.2. Related Work

With the continuous advancement of urbanization, the construction of urban sidewalks has evolved, exhibiting distinct characteristics across different periods. The materials and designs used in these constructions are notably diverse. While extensive research has been conducted on skid resistance mechanisms, as well as the relationship between pavement materials and skid resistance [23,24,25,26,27], the selection and application of paving materials for pedestrian spaces differ significantly from those used for driveways. Kim et al. [28] employed a dynamic friction tester and a laser-scanning confocal microscope to measure the skid resistance between four types of metal floors and three shoe samples. By calculating the two-dimensional parameters of floor texture, they proposed a theory and concept of floor surface wear, revealing that floor wear significantly impacts skid resistance performance. In China, the “Building Ground Engineering Anti-slip Technical Regulations” [29] classify ground skid resistance performance into four levels based on measurements from a tilting friction meter (BPT, British Pendulum Test) for wet surfaces and static friction coefficients (COF) for dry surfaces. Similarly, international standards such as the Australian standard [30] (AS 4586:2013), ASTM standard [31], and European standard [32] (DIN EN 16165:2021) have introduced an oil-wet inclined platform test. This test is conducted in both barefoot and shod conditions to simulate the skid resistance experienced by pedestrians walking on inclined surfaces under wet conditions. These methodologies aim to provide effective evaluations of the skid resistance levels of sidewalks.

Demarchi et al. [16] employed multiple methods, including the Tortus test, slope test, British Pendulum Test (BPT), and self-developed testing techniques, to evaluate the skid resistance of ceramic floor tiles with varying roughness. They compared the test results with empirical assessments of roughness and smoothness conducted by humans, revealing that the outcomes from most methods did not align with human perceptions of roughness and smoothness. Notably, the results of different testing approaches for measuring the coefficient of friction (COF) exhibited significant discrepancies. Walu et al. [17] the British Pendulum Number (BPN) to analyze the skid resistance of corridor surfaces and stairs in utility buildings. They compared the COF of different sole materials across various locations in public spaces and determined the impact of surface wear on skid resistance performance, particularly in terms of increased skid risk. Additionally, a tribometer was utilized to assess the skid resistance of pavement surfaces [33,34], with a focus on evaluating the performance of different sole patterns. Ma et al. [35] investigated various sidewalk materials, analyzing their prices, compositions, and water permeability. However, their study only briefly mentioned that skid resistance should meet the requirement of BPN ≥ 60, without thoroughly considering or addressing skid resistance performance. Hossain et al. [36] used the T2GO device, manufactured by ASFT (Swedish Airport Surface Friction Tester), to measure the COF of parking lots and sidewalks at the University of Waterloo in Ontario, Canada. They assessed the slip risk of each test section through subjective perception, providing a practical evaluation of skid resistance in real-world conditions.

Most existing research focuses on the material level, relying on micro-scale testing that is insufficient for evaluating the safety and security of walking or cycling paths. Such testing primarily determines whether individual paving materials or bricks meet the required specifications. However, factors such as the combination of materials, surface textures, geometric splicing relationships, and water penetration conditions can directly contribute to inadequate skid resistance and inconsistencies in performance. These issues can compromise safe riding speeds and increase the risk of accidents. Moreover, commonly used methods like the British Pendulum Number (BPN) and friction meter tests are subject to significant human and equipment variability, which undermines the accuracy of the results in reflecting the dynamic skid resistance of pavement spaces [37,38]. As a result, there is a pressing need for advanced and practical continuous testing methodologies to provide comprehensive data for evaluating the safety of walking and cycling paths. In contrast to research on skid resistance for non-motorized and pedestrian pavements, studies on motorized pavements and their associated non-contact testing methods are considerably more [39,40,41,42,43,44,45]. This field benefits from the use of relatively homogeneous paving materials, which simplifies testing and standardization. Motorized and high-traffic environments demand automated and efficient carriageway inspection systems. Non-contact inspection techniques, such as photography and LightLaser Detection and Ranging (LiDAR), have emerged as key tools for this purpose. Remote sensing data typically lacks the resolution required for detailed pavement analysis. In addition, street view data were utilized to provide a more practical and accessible solution for evaluating sidewalk conditions [46,47].

To comprehensively evaluate the skid resistance of multifactorial paving for non-motorized and pedestrian pavements—considering factors such as material type, morphological composition, and surface characteristics—this study examined ten sidewalks in Shanghai constructed with diverse materials widely used in China. A laser scanner was employed to capture detailed pavement surface data, enabling the extraction of key characteristics such as texture, seam width, and depth through advanced data processing techniques. Additionally, a portable continuous friction coefficient tester, T2GO, was used to measure the skid resistance of the sidewalks under both dry and wet conditions. The study analyzed the influence of surface texture, material, and joint design on skid resistance performance, aiming to establish a theoretical foundation for the design and management of pedestrian traffic facilities.

## 2. Methodology

### 2.1. Data Acquisition

T2GO was used to collect the skid resistance values of the test section in the dry state, and markers were made at the starting and ending points; then, within the starting and ending points, three points were selected at intervals of 0.5 m, the surface profiles of the three test points were obtained by laser scanning using the Gocator 2350; finally, each point was sprayed with 10 L of water to a length of 30 cm, and the skid resistance values of the three test points in the wet state were then tested using T2GO. The temperature distribution of the tested road surface was in the range of 10–15 °C, the T2GO test speed was 3–4 m/s, and the low-speed data were removed.

**Figure 1:**
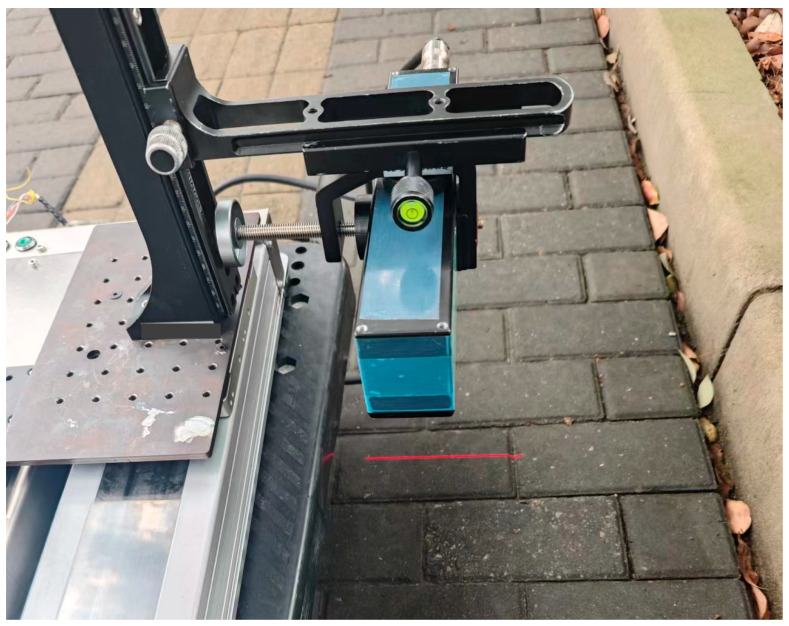
Acquisition of pavement surface state by laser scanner.

The Gocator 2350 laser scanner (LMI TECHNOLOGIES INC., Burnaby, BC, Canada) is a high-precision linear laser scanner, with a resolution reaching up to 0.15 mm in the X direction and 0.019 mm in the Z direction. The resolution in the Y direction is influenced by the moving speed and sampling rate of the laser scanner and is set to be consistent with the X direction. The detection accuracy of the laser scanning device enables the effective collection of road texture data, including the microscopic texture of the road surface [45], and the scanning process is presented in Figure 1.

**Figure 2:**
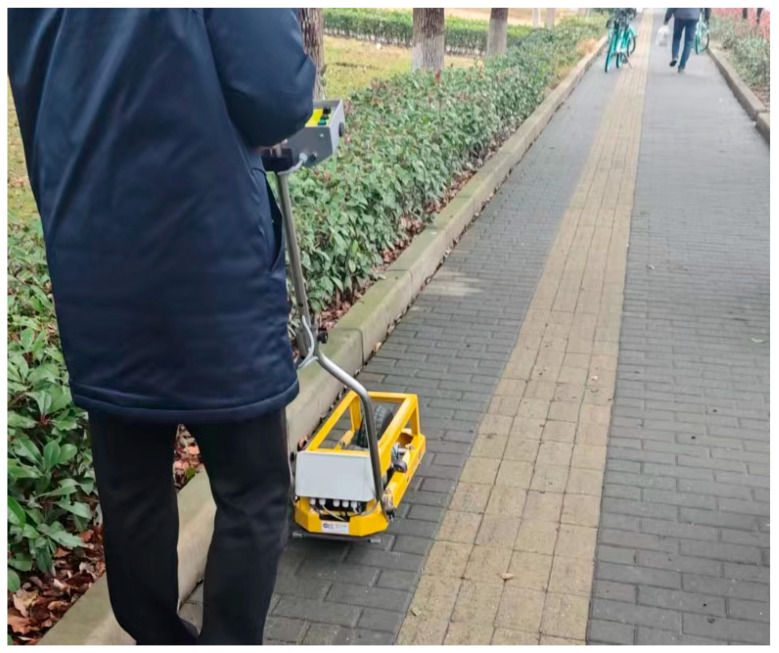
Friction coefficient measured by T2GO.

The T2GO continuous friction coefficient tester is capable of measuring the friction coefficient of the target area under both dry and wet conditions by manually holding the device and calculating the measured values following ASTM and EN standards, with high reliability [32,33], as depicted in Figure 2.

Based on a survey of common sidewalk paving types, a survey of common sidewalk paving types was conducted, and the experimental sites included two sidewalks on campus and four in urban areas, totaling 10 sections with a mean length of 50 m and a total of 250 positions. These materials exhibit diverse surface textures, drainage properties, and wear resistance, enabling a comprehensive evaluation of skid resistance mechanisms across material categories. These ten sidewalk types were classified into 6 grades according to their material absorption rates and surface water discharge rates at the outlet. The grading system reflects water drainage performance: the more efficiently water is eliminated within a shorter period, the higher the grade. Pavement water absorption, or permeability, was categorized into grades 1 to 6 (with grade 6 representing the highest permeability), as detailed in Table 1.

### 2.2. Construction of Surface Morphology Metrics

**Figure 3:**
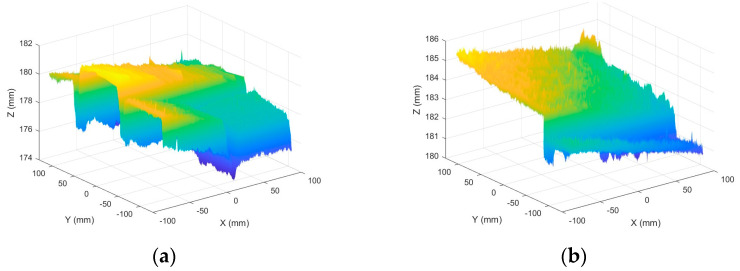

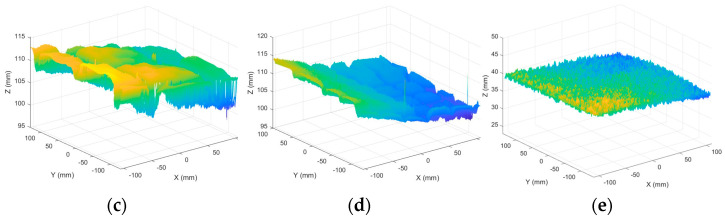
Surface morphology of different types of sidewalks: (**a**) permeable brick (#1); (**b**) stone (#3); (**c**) tile (#6); (**d**) decorative brick (#8); (**e**) fine mixture (#10).

MATLAB software 2023b was used to analyze the surface morphology data obtained by laser scanning, and the surface morphologies of different types of sidewalks are shown in Figure 3.

As illustrated in Figure 3, the surface of Footpath 1 exhibits several uneven areas, with the bricks not aligned in a strictly parallel manner. In contrast, the tiles on Pavement 2 and Pavement 4 demonstrate relatively parallel alignment. The surfaces of Sidewalk 3 and Sidewalk 6 were artificially padded to address the inherent unevenness of the ceramic material. The construction of Sidewalk 5 was notably more complex, as each tile varied in size and shape, leading to an increased number of joints across the surface. Overall, continuous laser scanning proves to be an effective method for measuring and reconstructing the complete morphological characteristics of the paving material assemblage.

To accurately characterize the complexity of surface texture and joint conditions, this study employs the mean texture depth (*MTD*) to represent the depth of the surface texture of the materials used. The fractal dimension (*FD*) is utilized to quantify the horizontal complexity of the surface texture, while a power spectral density function is applied to distinguish between macroscopic (*HL_TX_*) and microscopic (*WL_TX_*) texture levels. High texture density often indicates regions with more complex texture features, associated with greater detail and variation, while low texture density corresponds to more homogeneous areas with less variation in texture features. The micro-texture wavelength range was identified as [0, 0.5] mm, corresponding to a frequency range of [2, +∞] mm⁻^1^. Experimental analysis revealed that 38.46 mm⁻^1^ represents the optimal upper-frequency limit for micro-texture extraction, resulting in a refined frequency range of [2, 38.46] mm⁻^1^. This range effectively captures the predominant micro-texture wavelengths spanning [0.026, 0.5] mm. The calculation formulae for these metrics are derived from previous literature [45,46], as show in Equations (1)–(4).

(1) $$MTD=\frac{\sum_{k=1}^{N}\left(Z_{95}-Z_{ij}\right)}{W\times H},\mathrm{where}Z_{ij}<Z_{95},$$

where *Z*_95_ is the queried value of the 95th percentile *Z* coordinate of the textured point cloud data, *W* and *H* are the total number of pixels in the horizontal and vertical directions, respectively, *Z_ij_* is the height value corresponding to the pixel at (*i, j*), and *N* is the number of data under the *Z*_95_ plane.

(2) $$FD=\underset{n\to \infty }{lim}\frac{\log M(n)}{n\log2},$$

where *FD* is the fractal dimension of the curve (profile), and *M*(*n*) is the number of points where the box intersects the curve.

(3) $$HL_{TX}=L_{TX,0.5-\infty }=10\mathrm{lg}(\sum_{m=0.5}^{\infty }10^{\frac{L_{TX,m}}{10}}).$$

(4) $$WL_{TX}=L_{TX,0.026-0.5}=10\mathrm{lg}(\sum_{m=0.026}^{0.5}10^{\frac{L_{TX,m}}{10}}).$$

**Figure 4:**
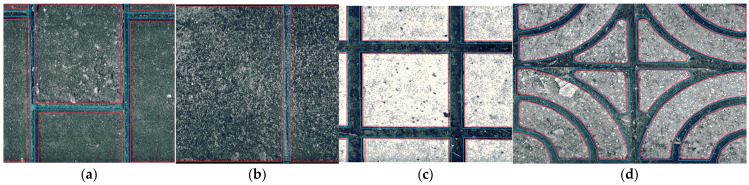
Calculated areas for different types of sidewalk pavements: (**a**) permeable brick; (**b**) stone; (**c**) tile; (**d**) decorative brick.

Additionally, calculations were performed for the mean depth (Depth) and area density (Density) of the joints within the scanned area of each pavement to characterize these features. The texture index was calculated exclusively for each brick within the pavement, as indicated by the area within the red border, while the seam index was calculated for the area within the blue border, as shown in Figure 4.

### 2.3. Correlation Analysis

To investigate the interrelationships between morphological indicators and skid resistance, a Pearson’s correlation analysis was first conducted to assess linear relationships between variables; the equation is shown in (5).

(5) $$PR=\frac{\mathrm{cov}\left(x,y\right)}{\sigma_{x}\sigma_{y}},$$

where *PR* is the element of the correlation matrix *S*. The meanings of *x* and *y* are the *x*th and the *y*th columns of the dimensionless matrix *X*. The *cov*(*x, y*) is the covariance between *x* and *y*, and $\sigma_{x}\;and\;\sigma_{y}$ are the standard deviations of *p* and *q*.

### 2.4. Hierarchical Regression Model Construction

Ordinary linear regression models assume that all observations are independently and identically distributed, thus disregarding the hierarchical structure of the data, which may result in inaccurate parameter estimates and underestimation of standard errors, consequently affecting the reliability of statistical inference. Therefore, a hierarchical regression model, which is a statistical model employed for the analysis of data characterized by a hierarchical or nested structure, was constructed in this study. Due to the limited availability of data, ordinary least squares (OLS) were utilized for parameter estimation.

Dependent variables include *Dμ__Dry_* (dry friction coefficient) and *Dμ__Wet_* (wet friction coefficient). Independent variables include *MTD*, *FD*, *HL_TX_* (macro-texture density), *WL_TX_* (micro-texture strength), density (seam density), and depth (seam depth). Standardization: All variables were Z-score standardized to eliminate the effect of magnitude. A two-stage hierarchical regression approach was adopted to evaluate the incremental contribution of joint parameters beyond texture indicators: Layer 1—texture indicators only (*MTD*, *FD*, *HL_TX_*, *WL_TX_*), layer 2—add seam parameters (density, depth).

The variance inflation factor (*VIF*) was utilized to detect the presence of multicollinearity in the model, i.e., whether there is a high degree of linear correlation between the independent variables, as in Equation (6).

(6) $${VIF}_{j}=\frac{1}{1-R_{j}^{2}},$$

where ${VIF}_{j}\;$ is the variance inflation factor of the independent variable *X_j_*, and $R_{j}^{2}$ is the determination coefficient by regression with *X_j_* for other independent variables as dependent variables.

Variance inflation factor (*VIF*) values were calculated for both dry and wet conditions to conduct a multicollinearity check for these independent variables. The larger the *VIF* value, the higher the degree of covariance between the independent variables, which may result in unstable estimation of the model parameters, affecting the model’s reliability and predictive ability. It is widely accepted that when the *VIF* value exceeds 10, a significant multicollinearity issue is present.

The determination coefficient (*R*^2^), incremental *R*^2^, and *p*-value were utilized to assess the fitting efficacy of the prediction model, and the mean absolute error (*MAE*) was selected to evaluate the prediction accuracy; these metrics were calculated according to Equations (7)–(9).

(7) $$R^{2}=1-\frac{\sum_{i=1}^{n}{\left(y_{i}-{\hat{y}}_{i}\right)}^{2}}{\sum_{i=1}^{n}{\left(y_{i}-\bar{y}\right)}^{2}},$$

(8) $${∆R}^{2}=R_{2}^{2}-R_{1}^{2},$$

(9) $$MAE=\frac{1}{n}\sum_{i=1}^{n}\left|y_{i}-{\hat{y}}_{i}\right|,$$

where n is the sample size of $y_{i}$, $y_{i}$ is the measured value of the dependent variable of the *i*th sample, $\bar{y}$ is the mean measured value of the dependent variable of the samples, and ${\hat{y}}_{i}$ is the predicted value of the dependent variable of the ith sample. $R_{1}^{2}$ denotes the goodness of fit for a set of independent variables (layer 1) and $R_{2}^{2}$ denotes the goodness of fit with the addition of the new independent variables (layer 2).

## 3. Results and Discussion

### 3.1. Friction Coefficient Results

**Figure 5:**
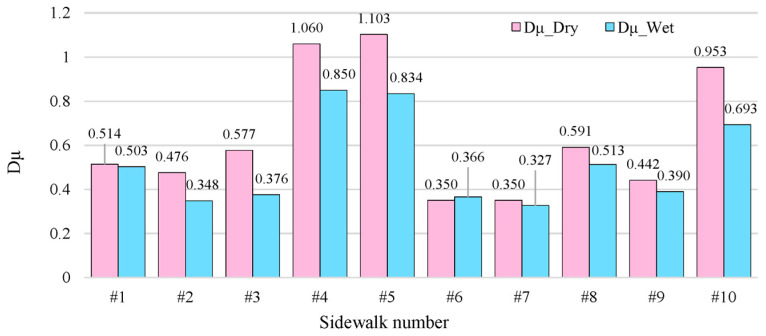
Friction coefficient measured results under dry and wet conditions.

The friction coefficients of the six kinds of sidewalks measured under dry and wet conditions are shown in Figure 5.

As demonstrated in Figure 5, several material types (brick #2, ceramic tiles #6 and #7) have wet friction coefficients that are lower than the minimum requirements of the national standard ’Anti-Sl Technical Regulations for Construction Ground Engineering’ (JGJ/T 331-2014). Consequently, it is imperative to eliminate hazardous products through the census, particularly in hospitals, schools, and other highly sensitive areas. It is also crucial to consider materials with a coefficient of friction reduction greater than 27% under wet conditions, as these materials exceed the safe redundancy range. The threshold compliance (patterned brick #9, wet friction of 0.390) materials also need attention. It is imperative to identify materials that breach the safety threshold and establish a census to implement a closed-loop management system (Early Warning—Intervention). This will prevent the accumulation of risk and ensure the safety of the environment.

In dry conditions, the skid resistance of the materials exhibited significant variations. Among the materials tested, stones #4 and #5 demonstrated the optimal performance, exhibiting a dry friction coefficient of 1.103 and 1.060, which approaches the ideal level of skid resistance (>1.0). This was followed by fine-grained colored mix #10, with a dry friction coefficient of 0.953. The three materials exhibited substantially higher skid resistance ratings compared to the other materials. Conversely, tiles #6 and #7 exhibited dry friction coefficients of 0.350, which is less than one-third of that observed for stone #5, demonstrating the weakest skid resistance. It is noteworthy that tile #8 exhibited an unusually high dry friction coefficient (0.591), suggesting the potential use of a special anti-slip process or surface treatment technology for tiles to achieve skid resistance.

The variation in friction performance under wet conditions is indicative of differences in the environmental adaptability of the materials. Stones #4 and #5 still demonstrate the highest skid resistance, with wet friction coefficients of 0.850 and 0.834, respectively. The attenuation rates are controlled at −19.8% and −24.4%, indicating that their microscopic pore structures are still effective in maintaining roughness under wet conditions. In contrast, drainage block #1 exhibited consistent performance, displaying only a modest decline in wet friction coefficient from 0.514 to 0.503 (attenuation rate −2.1%), indicative of its superior water resistance. Conversely, the wet friction coefficient of fine-grained mix #10 experienced a pronounced decrease from 0.953 to 0.693 (attenuation rate −27.3%), indicating a notable deficiency in its wetting performance.

Certain materials pose potential safety hazards in wet environments. For instance, the wet friction coefficient of tile #7 is as low as 0.327, which is already lower than the minimum standard (≥0.35) for ordinary pavements as stipulated in the ’Anti-sl Technical Regulations for Construction Ground Engineering’ (JGJ/T 331-2014); although patterned tile #9 only barely meets the standard (0.390), its limited anti-slip margin is not recommended for use in wet areas. What’s unusual is that tile #6 exhibited an increase in the coefficient of friction under wet conditions, measuring 4.6% higher than the standard, possibly due to surface contaminants being washed away by water flow.

Concerning wet and dry performance decay rates, drainage tile #1 (−2.1%) and stone #4 (−19.8%) demonstrated notable resistance to decay, while the substantial performance degradation exhibited by stone #3 (−34.8%) and fine-grained mix #10 (−27.3%) is a cause for concern. Material types demonstrate regular differences: the overall attenuation rate of the stone category ranges from −19.8% to −34.8% (#3 outliers have a detrimental effect on the overall performance), while the ceramic tile category shows a fluctuation of −13.2% to +4.6% due to variations in workmanship.

Concerning compliance with engineering code regulations, the wet friction coefficients of tile #7 (0.327) and stone #3 (0.376) did not meet the barrier-free access standard (≥0.45), and only stone #4/#5, tile #8, and fine-grained mix #10 met the standard for the high-risk areas with slopes >8% (≥0.55). The suboptimal performance of stone #3 could be attributed to its polished surface or low porosity.

### 3.2. Surface Morphology Index Results

#### 3.2.1. Surface Texture Results

**Figure 6:**
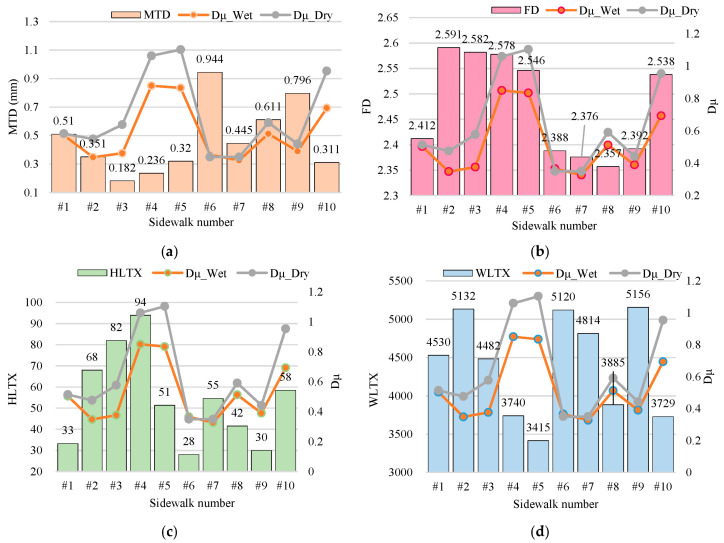
Results of sidewalk surface texture indexes: (**a**) *MTD*; (**b**) *FD*; (**c**) *HL_TX_*; (**d**) *WL_TX_*.

The mean *MTD*, *FD*, *HL_TX_*, and *WL_TX_* calculated results were drawn together with the Dμ results for the 10 sidewalks, as shown in Figure 6.

In the context of analysis Figure 6a, the correlation between *MTD* and friction performance is characterized by a certain degree of complexity. In dry conditions, certain materials (e.g., drain brick #1, *MTD* = 0.51, *Dμ__Dry_* = 0.514) demonstrate that texture depth may exert a positive influence on the coefficient of friction. However, the performance of tile #6 (*MTD* = 0.944, Dry = 0.350) and stone #5 (*MTD* = 0.32, *Dμ__Dry_* = 1.103) suggests that microstructure may have a more significant effect. The role of *MTD* is further obscured under wet conditions, as evidenced by the comparison between drain brick #1 (*MTD* = 0.51, *Dμ__Wet_* = 0.503) and tile #6 (*MTD* = 0.944, *Dμ__Wet_* = 0.366), which suggests that water permeability may be a pivotal moderator of wet skid resistance. Consequently, the skid resistance contribution of *MTD* must be evaluated in conjunction with the water permeability and microstructure of the material in question.

As shown in Figure 6b, the higher the *FD* value (high texture complexity), the better the *Dμ__Dry_*. For example, #5 (*FD* = 2.546, *Dμ__Dry_* = 1.103) and #4 (*FD* = 2.578, *Dμ__Dry_* = 1.060) performed the best. The exception was waterproof tile #2 (*FD* = 2.591, *Dμ__Dry_* = 0.476,) whose performance was limited due to a low *MTD* (0.351), suggesting that *FD* needs to be synergized with a reasonable *MTD*.

The available data (see Figure 6c,d) indicate the higher the *WL_TX_* value (the denser the micro-texture), the worse the *Dμ__Wet_*. For example, the high *WL_TX_* samples (#2: 5132, *Dμ__Wet_* = 0.348; #9: 5156, *Dμ__Wet_* = 0.390) have the lowest performance; the low *WL_TX_* samples (#5: 3415, *Dμ__Wet_* = 0.834; #4: 3740, *Dμ__Wet_* = 0.850) have the best performance. High *HL_TX_* (and possibly macroscopic texture complexity) may be unfavorable when wet, possibly because excessively high *HL_TX_* (>80) results in disorganized drainage paths (e.g., #3, *HL_TX_*~ = 82, *Dμ__Wet_* = 0.376). Scale characteristics of the texture may require more complex multi-scale analysis in conjunction with specific material types.

The variability in results underscores the importance of selecting appropriate measurement methods based on pavement type and conditions. Portable friction testers offer real-time friction measurements but can be influenced by surface irregularities, such as joints and seams. In contrast, laser scanners provide detailed 3D surface profiles and excel at capturing microscopic textures. Laser scanning technology enables the precise detection of pavement texture, joints, and geometric features through high-resolution 3D surface modeling.

#### 3.2.2. Seam Condition Results

**Figure 7:**
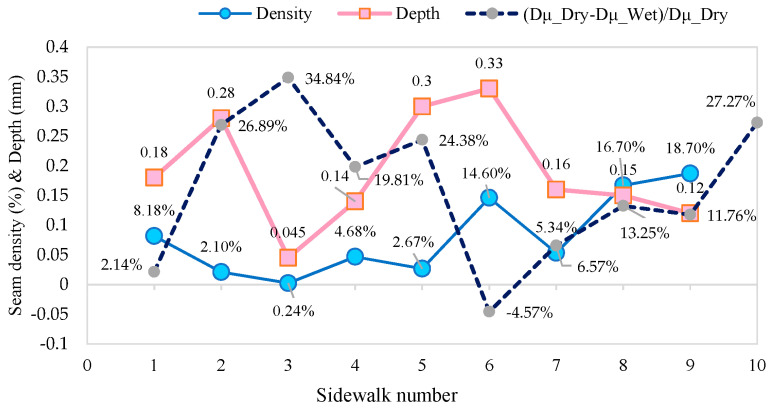
Joint condition results of sidewalks.

The calculated joint condition results and the Dμ reduction rate with the wet sidewalks are shown in Figure 7.

As shown in Figure 7, the effect of joint area percentage on the anti-slip properties of the materials demonstrated a significant environmental sensitivity, with data indicating that high joint percentages (>10%) generally result in reduced friction coefficients under wet conditions, as evidenced by materials #6 (14.6%, wet friction 0.366) and #9 (18.7%, wet friction 0.390). This suggests that excessive joints may divide the effective friction contact surface. However, outlier #8 (16.7%, wet friction 0.513) demonstrates that the detrimental effect of high percentages can be mitigated to a certain extent by incorporating anti-slip seam design elements, such as toothed groove structures or rough coatings. Conversely, Seamless material #10, despite its superior drying performance (0.953 dry friction), exhibited a substantial attenuation of its wetting performance of 27.3%, underscoring the significance of moderate seams (2–5% recommended) to facilitate drainage. The core conclusion drawn from this analysis is that seams should be maintained at less than 5% and that functional design should be employed to augment the skid resistance contribution of the seams themselves.

Joint depth plays a critical role in drainage efficiency and wet skid resistance. Deeper joints (≥0.25 mm) can enhance drainage by forming effective water channels, but their performance is closely tied to the material’s micro-textural strength (*WL_TX_*). For instance, material #5 (joint depth: 0.30 mm, *WL_TX_* = 3415) achieves a high wet friction coefficient (0.834) due to its sufficient joint depth and low *WL_TX_* (<3500), which promotes rapid water drainage. In contrast, material #2 (joint depth: 0.28 mm, joint density = 2.1%, *WL_TX_* = 5132) exhibits poor drainage despite the similar depth, as its high *WL_TX_* (indicating dense micro-textures) impedes water penetration, resulting in a low wet friction coefficient (0.348). Shallow joints (<0.15 mm) pose significant risks. For example, material #9 (depth: 0.12 mm) fails to redirect water flow, leading to surface water retention and a reduced wet friction coefficient (0.390). To optimize performance, joint depth should be tailored to environmental conditions. In standard areas, a depth of 0.15–0.25 mm paired with moderate permeability (e.g., material #1, depth: 0.18 mm, wet friction: 0.503) is recommended. In rain-prone or sloped regions, deeper joints (≥0.25 mm) combined with low *WL_TX_* materials (<3500) are preferable.

The interaction between joint density and depth further influences outcomes. High joint density (>10%) with shallow depth (e.g., #9: 18.7% density +0.12 mm depth) creates high-risk scenarios, yielding the lowest wet friction (0.390). Conversely, low density with deep joints (e.g., #5: 2.67% density +0.30 mm depth) achieves superior skid resistance (0.834). A balanced design (e.g., #1: 8.18% density +0.18 mm depth) offers optimal performance in typical conditions. In addition, integrating *WL_TX_* into joint evaluation is essential. High *WL_TX_* materials require increased depth (≥0.25 mm) to compensate for their limited drainage capacity, whereas low *WL_TX_* materials (e.g., porous stone) allow moderate depth reduction (0.15–0.20 mm) for cost efficiency without compromising safety. This approach underscores the need to harmonize joint geometry with micro-textural properties, ensuring reliable pavement performance across diverse environmental conditions.

### 3.3. Correlation Analysis Results

**Figure 8:**
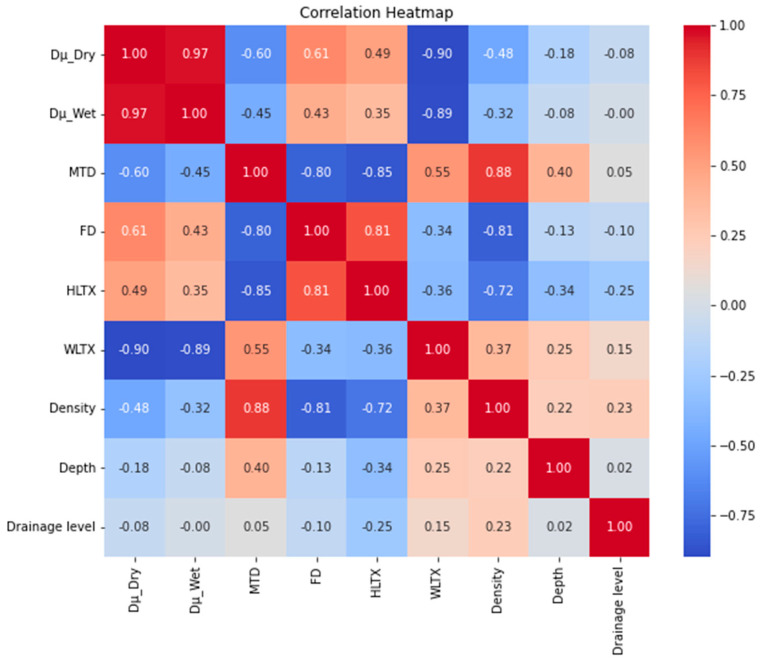
Correlation map for sidewalk surface morphological indicators and friction coefficient.

The Spearman correlation coefficients for the indicators, including dry and wet dynamic friction coefficients (Dμ-D and Dμ-W), *MTD*, *FD*, *HL_TX_*, *WL_TX_*, joint width, depth, and drainage level, were calculated, and the results were drawn as a heatmap, as shown in Figure 8.

Figure 8 shows that dry and wet friction properties are highly synchronized, with excellent material performance in dry conditions usually predicting stability in wet conditions. *MTD* is moderately negatively correlated (r = −0.60) with the coefficient of dry friction (*Dμ__Dry_*) and weakly negatively correlated (r = −0.45) with the coefficient of wet friction (*Dμ__Wet_*). High *MTD* value (>0.5 mm) materials (e.g., #6, *MTD* = 0.944, *Dμ__Wet_* = 0.366) showed a significant performance degradation due to the reduction in effective contact area caused by excessive roughness. *FD* was positively correlated with the coefficient of dry friction (*Dμ__Dry_*) (r = 0.61) and weakly positively correlated with the coefficient of wet friction (*Dμ__Wet_*) (r = 0.49). High *FD* (>2.5) materials (e.g., #1, *FD* = 2.412, *Dμ__Dry_* = 0.514) enhance dry friction through complex texture, but wetting performance is dependent on water permeability (e.g., #1, *WL_TX_* = 4530). Density, depth, and drainage level have a low correlation with the coefficient of friction (|r|<0.3) but affect performance through indirect pathways. High *HL_TX_* values (>80) materials (e.g., #3, #4) generally had lower coefficients of friction (*Dμ__Wet_* ≤ 0.376), suggesting that complex macro-textures may reduce the effective contact area. *WL_TX_* showed a significant negative correlation with the wetting coefficient of friction (e.g., #6, *WL_TX_* = 5120, *Dμ__Wet_* = 0.366; #5, *WL_TX_* = 3415, *Dμ__Wet_* = 0.834). In addition, the interaction of texture metrics should also be looked at, with low *WL_TX_* (<3500) tolerating high *MTD* (0.3–0.5 mm), e.g., #5 (*WL_TX_* = 3415, *MTD* = 0.32), and high *WL_TX_* (>4500) strictly limiting *MTD* to ≤0.2 mm (e.g., #6, *MTD* = 0.944, performance failure). Medium *HL_TX_* (40–60) + medium *WL_TX_* (3500–4000) is the best combination of anti-slip measured.

### 3.4. Hierarchical Regression

For dry conditions, *VIF* < 5 indicated no severe multicollinearity (e.g., the correlation between *MTD* and *FD*: r = 0.43). For wet conditions, *WL_TX_* and depth showed acceptable collinearity (*VIF* = 3.2).

The hierarchical regression analysis results are shown in Table 2. The obtained prediction models are shown in Equations (10) and (11).

(10) $$D\mu_{\_Dry}=0.61\cdot FD-0.53\cdot MTD+0.21\cdot Depth+\varepsilon .$$

(11) $$D\mu_{\_Wet}=-0.72\cdot WLTX+0.31\cdot Depth+\varepsilon .$$

The findings of the hierarchical regression analyses in Table 2 indicate that: (1) In the context of slip resistance in the dry state, *FD* exhibited a predominant role, functioning as the most robust predictor of dry slip resistance (*β* = 0.61), thereby suggesting that an increase in texture complexity is associated with an enhancement in *Dμ__Dry_*; conversely, *MTD* demonstrated a negative influence. This finding is consistent with the hypothesis that an excessively high *MTD* (>0.5 mm) may result in a reduction in the effective contact area due to excessive roughness (*β* = −0.53). Additionally, seam depth assistance was observed to slightly enhance performance with increased depth (*β* = 0.21), although the contribution was found to be limited (Δ*R*^2^ = 3%). (2) Concerning skid resistance in the wet state, *WL_TX_* dominated and was the core predictor of wet skid resistance (*β* = −0.76), with *WL_TX_* > 4500 significantly decreasing *Dμ__Wet_*; depth (*β* = 0.31 played a key role by contributing significantly (Δ*R*^2^ = 4%) to the drainage efficiency; and *FD*’s effect on skid resistance was weakened in the wet state (*β* = 0.18, *p* = 0.12).

The *MAE* values were 0.08 and 0.05 for the Dμ prediction models under dry and wet conditions, respectively, making the models manageable.

## 4. Conclusions

In light of the escalating concerns regarding the safety of sidewalks and cycle paths, along with the restricted availability of effective testing and assessment tools, this study delves into the factors influencing the skid resistance of various pavement surfaces under both dry and wet conditions. The skid resistance was measured by employing a T2GO device, while the surface texture morphology was captured through laser scanning using a Gocator 2350 laser scanner.

The research systematically explores the effects of drainage performance, seam conditions, and surface texture metrics on the friction coefficient in both dry and wet states. Moreover, the potential of utilizing pavement geometry to facilitate highly efficient non-destructive testing and accurate prediction of skidding performance was discussed. The key findings of this research are summarized as follows:

(1) Laser scanning enables detailed surface texture characterization, which is essential for understanding skid resistance mechanisms. The synergistic analysis of the T2GO friction test system with laser data provides the technical basis for predicting skid resistance in dynamic environments.
(2) Through hierarchical regression analysis, this study reveals that fractal dimension (*FD*) and mean texture depth (*MTD*) dominate skid resistance in dry environments, whereas microscopic texture strength (*WL_TX_*) and joint depth are the core influencing factors in wet environments, which provide quantitative guidance for safe pavement design and maintenance.
(3) Surface texture complexity (*FD*) and *MTD* are identified as dominant factors influencing dry-condition skid resistance, while drainage efficiency and seam design are critical in wet environments. Materials with *FD* > 2.5 and *MTD* <0.5 mm exhibit optimal dry skid resistance.
(4) Joints/seams need to be designed in conjunction with the microscopic densification properties of the material. A balanced design of density, depth, and *WL_TX_* offers optimal wet skid resistance.
(5) The preliminary design recommendations are as follows: High *FD* (>2.5), medium *MTD* (0.3–0.5 mm) materials should be selected for dry environments (e.g., stone #4, #5); in wet environments, *WL_TX_* <3500, Depth ≥0.25 mm (e.g., #5) should be prioritized, with high *WL_TX_* tiles (#6, #7) being avoided. Furthermore, it is recommended that seam density to be controlled less than 5%.

The T2GO + Gocator 2350 system was found to be a viable method of collecting typical sample data without impacting pedestrian traffic. Furthermore, simultaneous wet/dry testing helped to simulate real-world environmental changes. It has been proven that laser scanning captures micro-textural details that cannot be captured by traditional methods such as BPN, allowing for the identification of macro- and micro-features that affect surface drainage. However, the testing efficiency of existing high-precision laser scanners may limit their scalability. Consequently, further optimization of collection equipment and low-cost automated retrofitting based on well-defined metrics is required to enable large-scale urban data collection.

With the goal of a safe census, future research should prioritize expanding the dataset and refining methodologies to enhance the robustness of findings. A key focus should be on optimizing sensor deployment and data extraction processes to improve the efficiency and scalability of pavement monitoring systems. This includes refining algorithms for texture analysis and integrating multi-sensor data to enable comprehensive evaluations.

To clarify the feasibility of shared paved spaces for bikes/wheelchairs, pavement contact modeling through finite element analysis could be employed to develop safe speed models tailored to different riding conditions.

## Figures and Tables

**Table 1:** Summary of experimental sites and material types.

No.	Sites	Pavement Type	Drainage Level	Photograph
#1	Yumai Road	Permeable brick 1	6	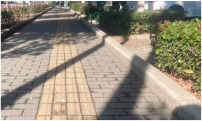
#2	Tongji Campus	Permeable brick 2	4	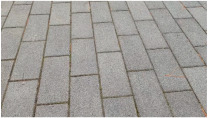
#3	Wuning Road	Stone 1	2	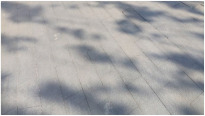
#4	Tongdaoyou	Stone 2	3	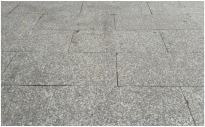
#5	Xiaoban	Stone 3	3	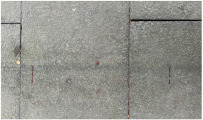
#6	Tongji Campus	Tile 1	1	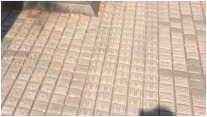
#7	Changshou Road	Tile 2	2	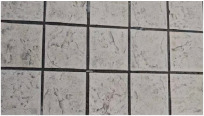
#8	Tongdaozuo	Tile 3	4	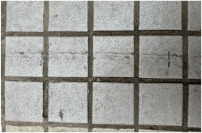
#9	Jiasongbei Road	Decorative brick	5	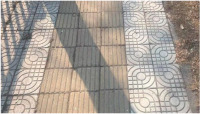
#10	Tushuqian	Color fine mixture	2	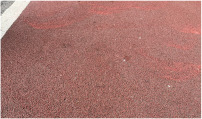

**Table 2:** Friction coefficient prediction model analysis results.

Predicted Variable	Stage	*R* ^2^	∆*R*^2^	Significant Variables (*β*)	*p*
*Dμ__Dry_*	1 (Texture)	0.72	-	*FD (β = 0.61), MTD (β = −0.53)*	<0.01
	2 (Seam)	0.75	0.03	*FD (β = 0.58), Depth (β = 0.21)*	Depth: 0.04
*Dμ__Wet_*	1 (Texture)	0.85	-	*WL_TX_ (β = −0.76), FD (β = 0.18)*	<0.01
	2 (Seam)	0.89	0.04	*WL_TX_ (β = −0.72), Depth (β = 0.31)*	Depth: 0.008

## Data Availability

The data presented in this study are available on request from the corresponding author. The data are not publicly available due to the confidentiality requirements for unfinished research projects.
